# *In Vitro* Disease Modeling of Hermansky-Pudlak Syndrome Type 2 Using Human Induced Pluripotent Stem Cell-Derived Alveolar Organoids

**DOI:** 10.1016/j.stemcr.2019.05.022

**Published:** 2019-07-09

**Authors:** Yohei Korogi, Shimpei Gotoh, Satoshi Ikeo, Yuki Yamamoto, Naoyuki Sone, Koji Tamai, Satoshi Konishi, Tadao Nagasaki, Hisako Matsumoto, Isao Ito, Toyofumi F. Chen-Yoshikawa, Hiroshi Date, Masatoshi Hagiwara, Isao Asaka, Akitsu Hotta, Michiaki Mishima, Toyohiro Hirai

## Main Text

(Stem Cell Reports *12*, 431–440; March 5, 2019)

In the originally published version of our manuscript, the legend to Figure S3 in the Supplemental Information was accidentally removed during the proofing process. To address this, we have now replaced the Supplemental Information with the correct version. We apologize for the oversight and for any resulting confusion.

